# Supplementary material for: Transgenerational Epigenetic and Phenotypic Inheritance Across Five Generations in Sheep
Source: Int J Mol Sci. 2025 Jul 3;26(13):6412. doi: 10.3390/ijms26136412 (PMC12250259; doi:10.3390/ijms26136412)
Supplement: Supplementary file 1 [file ijms-26-06412-s001.zip › Supplementary_Table S1.pdf]

Supplementary Table S1. Number of overlapping genes across different generations

Generation	DMGs
F0-F3	163
F0-F4	102
F0-F1-F3	71
F0-F1-F4	41
F0-F1-F2-F4	26
F1-F2	278
F1-F3	113
F1-F4	67
F1-F2-F3	59
F1-F2-F4	37
F2-F3	137
F2-F4	92
F2-F3-F4	33
F3-F4	114
